# Study Protocol - Accurate assessment of kidney function in Indigenous Australians: aims and methods of the eGFR Study

**DOI:** 10.1186/1471-2458-10-80

**Published:** 2010-02-19

**Authors:** Louise J Maple-Brown, Paul D Lawton, Jaquelyne T Hughes, Suresh K Sharma, Graham RD Jones, Andrew G Ellis, Wendy Hoy, Alan Cass, Richard J MacIsaac, Ashim K Sinha, Mark AB Thomas, Leonard S Piers, Leigh C Ward, Katrina Drabsch, Sianna Panagiotopoulos, Robyn McDermott, Kevin Warr, Sajiv Cherian, Alex Brown, George Jerums, Kerin O'Dea

**Affiliations:** 1 Menzies School of Health Research, Institute of Advanced Studies, Charles Darwin University, Darwin, Australia; 2 Division of Medicine, Royal Darwin Hospital, Darwin, Australia; 3 Chemical Pathology, St Vincent's Hospital, Sydney, Australia; 4 University of Melbourne, Department of Medicine, Austin and Northern Health, Heidelberg, Victoria, Australia; 5 Centre for Chronic Disease, The University of Queensland, Australia; 6 The George Institute for International Health, University of Sydney, Sydney, Australia; 7 Department of Endocrinology, Endocrine Centre Austin Health & University of Melbourne, Heidelberg Repatriation Hospital, Heidelberg West, Victoria, Australia; 8 Endocrine and Diabetes Unit, Cairns Base Hospital, Queensland, Australia; 9 Department of Nephrology, Royal Perth Hospital, Perth, Australia; 10 Centre for Health and Society, School of Population Health, University of Melbourne, Melbourne, Australia; 11 School of Chemistry and Molecular Biosciences, The University of Queensland, Australia; 12 Sansom Institute for Health Research, UniSA, Adelaide, Australia; 13 Department of Renal Medicine, Alice Springs Hospital, Alice Springs, Australia; 14 Baker IDI Heart and Diabetes Institute, Alice Springs, Australia

## Abstract

**Background:**

There is an overwhelming burden of cardiovascular disease, type 2 diabetes and chronic kidney disease among Indigenous Australians. In this high risk population, it is vital that we are able to measure accurately kidney function. Glomerular filtration rate is the best overall marker of kidney function. However, differences in body build and body composition between Indigenous and non-Indigenous Australians suggest that creatinine-based estimates of glomerular filtration rate derived for European populations may not be appropriate for Indigenous Australians. The burden of kidney disease is borne disproportionately by Indigenous Australians in central and northern Australia, and there is significant heterogeneity in body build and composition within and amongst these groups. This heterogeneity might differentially affect the accuracy of estimation of glomerular filtration rate between different Indigenous groups. By assessing kidney function in Indigenous Australians from Northern Queensland, Northern Territory and Western Australia, we aim to determine a validated and practical measure of glomerular filtration rate suitable for use in all Indigenous Australians.

**Methods/Design:**

A cross-sectional study of Indigenous Australian adults (target n = 600, 50% male) across 4 sites: Top End, Northern Territory; Central Australia; Far North Queensland and Western Australia. The reference measure of glomerular filtration rate was the plasma disappearance rate of iohexol over 4 hours. We will compare the accuracy of the following glomerular filtration rate measures with the reference measure: Modification of Diet in Renal Disease 4-variable formula, Chronic Kidney Disease Epidemiology Collaboration equation, Cockcroft-Gault formula and cystatin C- derived estimates. Detailed assessment of body build and composition was performed using anthropometric measurements, skinfold thicknesses, bioelectrical impedance and a sub-study used dual-energy X-ray absorptiometry. A questionnaire was performed for socio-economic status and medical history.

**Discussion:**

We have successfully managed several operational challenges within this multi-centre complex clinical research project performed across remote North, Western and Central Australia. It seems unlikely that a single correction factor (similar to that for African-Americans) to the equation for estimated glomerular filtration rate will prove appropriate or practical for Indigenous Australians. However, it may be that a modification of the equation in Indigenous Australians would be to include a measure of fat-free mass.

## Background

Indigenous Australians have rates of cardiovascular disease (CVD) mortality some 7-10 times higher than non-Indigenous Australians aged 25-64 years, a prevalence of diabetes some 10 times higher (age 20-50 years), new cases of end stage kidney disease (ESKD) 10-15 times higher and life expectancy 15-20 years shorter [1-3]. Type 2 diabetes is common amongst Indigenous Australians with ESKD: 77% of Indigenous Australians with new ESKD in 2007 compared to 33% of non-Indigenous Australians have Type 2 diabetes as a comorbidity [4]. Rates of ESKD in Indigenous Australians are disproportionately highest in those in central and northern Australia [5]. This group of people is widely dispersed and heterogenous: they live in urban, regional and remote settings and have wide variation in diet, body habitus, genetic admixture and socioeconomic background [6-8].

With the serious burden of ESKD in Indigenous Australians it is essential to be able to assess accurately renal function in these populations. Glomerular filtration rate (GFR) is the best overall marker of renal function in people with healthy or diseased kidneys [9] and is an independent predictor of renal and cardiovascular events [10,11]. At present there are no validated methods for estimating GFR in Indigenous Australians [12,13]. Differences in body build and body composition between Indigenous and non-Indigenous Australians suggest that creatinine-based estimates of GFR (eGFR) derived for European populations may not be appropriate for Indigenous Australians, as muscle mass is a key determinant of serum creatinine levels. Compared to Australians of European background, Indigenous Australians from remote central and northern Australia have more fat for a given weight or BMI [7].

It is increasingly recognised that current creatinine-based formulae used to estimate GFR might not be generalisable across all clinical presentations [14,15]. They also lack validation in ethnic populations apart from Caucasians and African Americans [12,14]. The Modification of Diet in Renal Disease 4-variable formula (MDRD) was derived from a study population with impaired renal function; and thus is less accurate within a "healthy range" GFR. To address this issue a revised equation (Chronic Kidney Disease Epidemiology Collaboration, CKD-EPI) has been described [16].

An alternate approach is to use a different analyte from creatinine to estimate renal function and develop GFR prediction equations. Serum cystatin C has been proposed as a simple, reliable and accurate marker of GFR when compared to creatinine-based methods [17,18]. Although there is some evidence to suggest that cystatin C levels might be influenced by extra-renal factors, numerous studies have suggested that cystatin C levels or GFR estimates based on cystatin C levels alone are more accurate indices of renal function than creatinine-based methods [19]. However, at this time cystatin C assays are not routinely available, they are considerably more expensive than creatinine assays and are not yet fully standardised. Thus for the immediate future, optimisation of creatinine-based methods remains important.

The aim of the present study is to evaluate and improve if necessary the accuracy and precision of eGFR estimates in Indigenous Australians, taking into account the heterogeneity in body build and body composition across different populations. The study's hypotheses are: (i) that differences in body build and body composition between Indigenous and non-Indigenous Australians will affect the validity of creatinine-based estimated measures of GFR (using existing predictive equations) and that accuracy of estimates of GFR will vary across different Indigenous population groups; and (ii) that a more accurate assessment of GFR will be obtained by a formula involving serum creatinine and percent fat free mass (obtained by a simple, validated bioimpedance measurement) or by using an alternative biochemical measure such as cystatin C. It is anticipated that this will be of use in both Indigenous and non-Indigenous Australians of variable body builds, particularly at GFR>60 ml/min/1.73 m^2^.

## Methods/Design

### Setting & location

Participants were recruited from 4 geographical regions: Top End (of Northern Territory, Australia), Central Australia, Far North Queensland and Western Australia. These regions were chosen as resident Indigenous Australians bear a disproportionately larger burden of ESKD than those from other regions of Australia. Within these four regions, the following Aboriginal Medical Services or health facilities were recruitment centres: Top End - Royal Darwin Hospital, Danila Dilba Health Services (Darwin), Bagot Community Health Centre (Darwin), Wurli Wurlinjang (Katherine), Ngalkunbuy Health Service (Miwatj Health Aboriginal Corporation, Galiwinku), Katherine District Hospital, Gove (Nhulunbuy) District Hospital; Central Australia - Alice Springs Hospital, Tennant Creek Hospital, Central Australian Aboriginal Congress, Anyinginyi Health Aboriginal Corporation (Tennant Creek), Amooguna Health Centre, Titjikala Health Centre; Western Australia - Bega Garnbirringu Health Service Aboriginal Corporation (Kalgoorlie), Kimberley Aboriginal Medical Services; Far North Queensland - Wuchopperen Health Service (Cairns), Thursday Island Hospital, Cairns Base Hospital Diabetes Centre, Meriba Dhoeynidhay Yabu Torres Strait & Northern Penninsula Area Community Council.

### Participants and recruitment

Participants were Indigenous Australians (target n = 600 with equal numbers of males and females) aged 16 years and above, from urban, rural and remote regions of Northern and Central Australia across the following 5 strata:

(i) "healthy" group: nil diabetes, hypertension, chronic kidney disease (CKD), albuminuria;

(ii) participants with diabetes or albuminuria & eGFR (MDRD-4) >90 ml/min/1.73 m^2^;

(iii) eGFR 60-90 ml/min/1.73 m^2^;

(iv) eGFR 30-59 ml/min/1.73 m^2^;

(v) eGFR <15-29 ml/min/1.73 m^2^.

Indigenous Australians fulfilled the definition of 'Aboriginal and/or Torres Strait Islander' according to the standard method used in National Census data collection: "1) is of Aboriginal and/or Torres Strait Islander descent; 2) identifies as an Australian Aboriginal and/or Torres Strait Islander; and 3) is accepted as such by the community in which he or she lives or has lived".

Exclusion criteria were: participants with rapidly changing kidney function, participants receiving dialysis, women who were pregnant or breastfeeding and people with a history of allergy or adverse reaction to iodine-based contrast media.

Participants with CKD and/or diabetes were recruited from participating Aboriginal Medical Services and the nephrology, endocrinology and outreach kidney disease clinics associated with Royal Darwin Hospital, Alice Springs Hospital, Cairns Base Hospital and the Royal Perth Hospital. The identification and recruitment of the "healthy" group was through community networks, staff members of health services, word-of-mouth, self referral and family members of participants.

### Funding

Major funding was provided by the National Health and Medical Research Council of Australia (NHMRC, project grant #545202). Prior to award of this grant, pilot funding was received from: Kidney Health Australia, the Colonial Foundation of Australia and Menzies School of Health Research. Additional funding for equipment was received from Rebecca L Cooper Medical Research Grant.

### Ethics

The study was approved by the joint Menzies School of Health Research - Northern Territory Department of Health and Community Services Human Research Ethics Committee. The project was considered and approved by both the Aboriginal sub-committee, which has absolute right of veto, and by the main committee. The study was also approved by the following Human Research Ethics Committees: Central Australian Human Research Ethics Committee, Western Australian Aboriginal Health Information and Ethics Committee, Royal Perth Hospital Ethics Committee and Cairns and Hinterland Health Services District Human Research Ethics Committee.

### Staff

Six people were employed on contracts over the course of the study. Of these, four were Indigenous and two were non-Indigenous. Additional people were employed on a casual basis during visits to discrete communities; these community members acted as local facilitators. One post-graduate research student (who is Indigenous) also participated in the study and was substantially involved in data collection, whilst also making a significant contribution to study profile and media relations. The majority of staff members had prior health qualifications and experience: three were Aboriginal Health Workers, two were registered nurses and the post-graduate research student was a specialist nephrologist. The staff member without a health qualification completed a venipuncture certification course.

### Consent

Potential participants were provided with written information about the study, face-to-face discussion with a staff member, and given an opportunity to ask questions. If required, an interpreter was employed to explain all information relating to the study. Those who indicated that they wished to participate were then asked to complete and sign a consent form. Consent was obtained using the NHMRC Guidelines for Ethical Conduct in Aboriginal and Torres Strait Islander Health Research. All participants were informed that their participation was voluntary and that they could refuse or withdraw from participating and need give no reasons nor justification for their decision and that it would not affect their medical care. A parent or guardian was asked to sign the form as well if the participant was under 18 years old. Participants were asked to give separate consent for various elements of the study, including: iohexol injection and blood and urine samples; body measurements, body composition tests, blood pressure and questionnaire; and collecting further information from their medical records. They were also asked whether they wanted a report sent to their primary health care provider and whether their blood and urine samples could be stored at Menzies School of Health Research for 5 years and used for future studies related to diabetes and kidney disease.

### Examination protocol

Following confirmation of contact details and eligibility and after obtaining informed consent, performance of the reference measure of GFR and a health examination was undertaken. This examination included: collection of blood and urine samples; clinical and anthropometric measurements; and the administration of questionnaires. The performance of the reference measurement of GFR required a minimum of 4 hours for participants, during which time participants were given a healthy meal and health promotion information. Details of the examination protocol:

(i) Reference measure of GFR: determined by measuring the renal clearance of non-isotopic iohexol. Iohexol was injected (5.445 ml, 300 mg/ml "Omnipaque") into an antecubital vein (or forearm/hand vein if unable to use antecubital vein) and flushed with 10 ml of normal saline. The volume of iohexol includes the prime volume (0.445 ml) in the tubing of the butterfly cannula used to inject the iohexol. Extravasation was assessed by clinical inspection and palpation of the injection site. If there was any evidence of extravasation then the study was suspended and the participant invited to return in 3-7 days for assessment. Using the contralateral arm, venous blood samples (5 ml) were collected for measurement of iohexol at 120, 180 and 240 min post injection. Participants were closely supervised for possible adverse reactions - whilst also receiving culturally appropriate education about chronic kidney disease, diabetes and/or healthy lifestyle.

Slope-intercept GFR was calculated from the formula:

Slope-intercept GFR (mL/min) = k × Iohexol dose (μg)/C0 (μg/mL) where k is the slope of the semilog plot of plasma Iohexol concentration versus time (plotted using 3 points, refer above), and C0 is the calculated Iohexol concentration at time zero (intercept). This value was multiplied by 1.73 and divided by the body surface area (BSA, calculated from the equation BSA = 0.20247 × height (metres)^0.725 ^× weight (kg)^0.425 ^[20]. The BSA-slope intercept GFR value (mL/min/1.73 m^2^) was corrected by the Brochner-Mortensen correction factor = (0.990778 × GFR) - (0.001218 × GFR^2^) [21] providing a reference GFR value (mL/min/1.73 m^2^).

(ii) Blood and urine measures:

a. Serum creatinine measurements: performed both locally and at a central laboratory (The Department of Laboratory Medicine, Austin Health).

b. Estimates of GFR/creatinine clearance (where S_cr _is serum creatinine concentration in μmol/L, age in years, weight in kg, eGFR in mL/min/1.73 m^2^):

i. The adjusted MDRD-4 variable formula (for creatinine measurements traceable to the IDMS method and reported in μmol/L)

eGFR = 175 × [(S_cr _× 0.0113)^-1.154^] × (age)^-0.203 ^× (0.742 if female) × (1.212 if African-American) [22]. The above African-American "correction factor" was not used for Indigenous Australians.

ii. The CKD-EPI formula [16]:

GFR = 141 × min(Scr × 0.0113/k, 1)^α ^× max(Scr × 0.0113/k, 1)^-1.209 ^× 0.993^Age ^× 1.018 [if female] × 1.159 [if black], where Scr is serum creatinine, k is 0.7 for females and 0.9 for males, ^α ^is -0.329 for females and -0.411 for males, min indicates the minimum of Scr/k or 1, and max indicates the maximum of Scr/k or 1. As the correction factor "if black" applies to African-Americans this was not used for Indigenous Australians.

iii. The Cockcroft-Gault (C-G) formula: [23]

Creatinine Clearance (mL/min) = { [140 - age] × weight/[S_cr _× 0.814]} × (0.85 if female) *also calculated using ideal body weight (IBW) according to Therapeutic Guidelines protocol when the patient is obese (BMI>30 kg/m^2^). IBW = 50 kg + 0.9 kg/each cm over 152 cm (- 4.5 kg if female).

c. Cystatin C: non-fasting blood sample for measurement of cystatin C levels collected on the morning of iohexol clearance measurements.

d. Bloods (non-fasting) were taken with the 120 minute venous sample for iohexol: HbA1c, UEC, lipids (total and HDL cholesterol), high sensitivity c-reactive protein were performed as standard clinical care and analysed by local care providers. Although a fasting blood sample would be preferable for assessment of lipids and creatinine it was not practical in the setting of a renal or diabetes clinic. We therefore assessed non-fasting total and HDL cholesterol and instructed participants not to consume meat on the day of the examination prior to measurement of serum creatinine. A subset of participants was asked when they last ate a meal containing meat. The light meal provided by study staff to participants was given after the blood sample for creatinine was taken. Additional serum (8 ml) was collected at 120 minutes for cystatin C, inflammatory markers and a stored sample.

e. Urine: sample collected for microscopy and culture and determination of the albumin to creatinine ratio (ACR), performed as standard clinical care and analysed by local care providers. Urine cotinine was measured for quantitative assessment of cigarette smoking (recent nicotine consumption).

(iii) Clinical and anthropometric measures:

a. Blood pressure was measured three times after the participant had been seated quietly for at least 5 minutes. A Welch Allyn Spot Vital Signs monitor (Welch Allyn Medical Products, Skaneateles Falls, USA) was used to measure systolic and diastolic pressure, with three minutes between readings. The pulse rate was recorded with each reading.

b. Height was measured to the nearest 0.1 cm using a wall-mounted stadiometer [24]. Participants were shoeless and wore light clothing. They were instructed to stand facing forward with weight distributed evenly on both feet, with heels together and against the wall and arms hanging loosely by their sides. The participant's head was positioned so that the Frankfort Plane was horizontal. The participant was then instructed to keep his or her eyes focused on a point straight ahead, breathe in deeply, and stretch to his or her fullest height, with heels still on the ground. The measuring plate was then lowered onto the scalp until it rested firmly on the top of the head and a reading to the nearest 0.1 cm was obtained and recorded. Following the measurement of weight (as described below), a second height measurement was obtained and recorded, again to the nearest 0.1 cm. If the two height measurements differed by more than 0.5 cm, a third measurement was taken and recorded.

c. Weight in kilograms was measured using a Seca digital portable scale (Model 767 and 841, Seca Deutschland, Hamburg, Germany). Participants were asked to remove shoes, heavy garments, heavy jewellery, belts, loose change, keys, mobile phones, and other items from pockets. With the scales at zero, participants were asked to stand on the centre of the base with feet together, arms hanging loosely at the sides and the head facing forward. Weight was recorded to the nearest 0.1 kilogram. Two separate measurements of weight were recorded and if the first two differed at all then a third measurement of weight was taken and recorded.

d. Waist and hip circumference were measured in centimetres using a 2-metre non-stretch flexible steel tape (Model W606PM Lufkin, Texas, USA). Following removal of outer clothing, tight-fitting garments, belts and heavy items from pockets, participants were asked to stand comfortably erect in a relaxed manner, breathing normally, with weight balanced evenly on both feet, feet about 25-30 cm apart, and arms folded across the chest. Waist and hip measurements were taken alternately, with a minimum of two measurements for each. All measurements were taken and recorded to the nearest 0.1 centimetre. If the first two measurements of either waist or hip circumference differed by more than 1.0 cm, then a third measurement was taken for waist or hip, as relevant. Waist measurements were taken directly on the participant's skin. Hip measurements were taken over the participant's clothing, unless it was too tight or too baggy, in which case the participant was asked to remove it. The waist was defined as the midway point between the iliac crest and the costal margin. These two landmarks were identified and marked using a felt tip pen, and the distance between them measured, with the midpoint marked. Once the tape was around the participant's body at the appropriate height and the tape was horizontal, the participant was asked to breathe out gently. The measurement was taken at the end of a normal expiration, with the tape pulled snug but not compressing the underlying soft tissue. If landmarks were unable to be identified (in the occasional very obese participant), waist measurements were omitted. The hip circumference was defined as the widest circumference over the buttocks and below the iliac crest. The hip circumference was measured at several positions (starting with the widest part of the buttocks when viewed from the side) and the widest circumference recorded. Fatty aprons were not included in the measurement of the hips. The measurement was taken with the tape in a horizontal position, and the tape was pulled to allow it to maintain its position without causing indentation.

e. Skinfold thickness assessment was performed using Holtain calipers (Holtain Ltd, Crosswell, Pembrokeshire, United Kingdom) at 4 sites bilaterally: peripheral (biceps, triceps) and central (subscapular, suprailiac) [25]. Bilateral skinfold measurements were performed in all participants to ensure a site availability of longitudinal follow up in the event of an arterio-venous fistula insertion in CKD participants. The skinfold site was marked using a felt-tip pen and each site was identified as follows: biceps, anterior surface of the biceps midway between the acromial process and the elbow (antecubital fossa); triceps, posterior surface of triceps at midpoint from the acromial process (the most lateral and superior aspect of the acromial head) to the olecranon; subscapular, 2.5 cm infero-medially along a 45 degree line from inferior angle of the scapula; suprailiac, 2.5 cm supero-medially along a 45 degree line from where the medial border of the iliac crest tips in to the pelvis. The skin fold was then firmly grasped by the thumb and index finger, using the pads at the tip of the thumb and finger, and the skin fold gently pulled away from the body. The caliper was placed perpendicular to the fold, on the site marked, dialled up, at approximately 1 cm below the finger and thumb. While maintaining the grasp of the skin fold, the caliper was released so that full tension was placed on the skin fold. The dial was read to the nearest 0.20 mm, 2 seconds after the grip was fully released. Three measurements were taken at each site were possible.

(iv) Whole body and segmental bioelectrical impedance measurements (BIA) were performed at a single frequency (50 kHz) using a four-terminal impedance plethysmograph (Model DF50, ImpediMed, Brisbane). At study sites where a multi-frequency bioimpedance spectroscopy instrument (BIS, Model SFB7, ImpediMed, Brisbane) was available, this was used. BIS technology allowed prediction of body fluid volumes (intra- and extracellular water as well as total body water) at the theoretically optimal frequencies, providing precision and accuracy not achievable using the simpler BIA device that operates at a single compromise frequency [26]. Prediction of body fluid volumes is particularly important in this study population at all stages of CKD - from hyperfiltration with "normal" renal function to ESKD (excluding dialysis). It is also worthy to note that BIS provides measures of both intra-and extracellular water spaces and since muscle mass is strongly related to intracellular water space this will provide the opportunity to assess whether predictive power can be increased by inclusion of this variable in GFR formulae.

BIA/BIS was not performed if a participant had an indwelling electrical device (such as cardiac pacemaker). Participants were asked to void (empty bladder) prior to the assessment. Assessment was performed after 5 minutes rest in the supine position with limbs abducted away from the trunk, ensuring that thighs were not touching. Eight electrodes (electrocardiograph-style gel electrodes) were placed on bilateral dorsal surfaces of hands and feet (two electrodes on each hand and foot); after the surface was cleansed with an alcohol wipe. At the wrist, the proximal electrode was placed in the midline of the ulnar styloid process and the distal electrode was placed 5 cm distal to the proximal electrode. At the ankle, the proximal electrode was placed at the midpoint between the medial and lateral malleoli and the distal electrode was placed 5 cm distal to the proximal electrode. Whole body and segmental impedance measurements were according to previously described methods [27].

(v) DXA Sub-study: Further assessment of body composition by dual-energy X-ray absorptiometry (DXA) assessment of total body fat mass, lean mass, bone mineral content and bone density was performed according to standard techniques in sites where this imaging technique was available: Darwin, Perth and Torres Strait. DXA is a reference method for assessing body composition but impedance methods, particularly inexpensive single frequency BIA, are much more practical in remote locations (where DXA is indeed not possible/available). Hence, the aim of the sub-study comparing DXA and BIA was to see if BIA is a reasonable substitute for DXA, thereby enabling body composition assessment in remote communities. In the present study all participants had body composition measured by BIA or BIS and anthropometry, and a subset (Darwin, Perth and Torres Strait) also had DXA measurements. The DXA data will be used to validate the impedance data.

(vi) Questionnaires:

*a. Medical history & medications - *self report and from medical records (where available) including assessment of cigarette smoking: current status, past history and self-reported quantity.

*b. Assessment of socio-economic status and related personal details *by brief questionnaire: usual place of residence, first language, language spoken at home, ethnicity of grandparents, income source, ever worked, education level, number of bedrooms and occupants in house, housing tenure and landlord type.

Feedback to participants was provided at the end of the examination. Brief intervention regarding diet, physical activity and health behaviours in particular smoking, was provided. Participants with unexpected abnormal results were referred immediately to the appropriate health care provider. In addition, written results were provided in a plain language and culturally appropriate manner to individual participants and to their nominated primary health care provider. Whenever possible, participants with abnormal results were first contacted by telephone or in person. The results packet was then mailed or hand-delivered, as appropriate. A $50 gift voucher to a local store was provided to participants with their results as a gift of appreciation for their participation in the study. Participants were not informed of this gift prior to their participation in the study and it was not used as an incentive to recruit participants to the study. The gift was agreed upon by study investigators after it was suggested by several key organisations in our community consultation: Kimberley Aboriginal Medical Services Council and Wuchopperen Health Service. Permission to give the gift was approved by all relevant Human Research Ethics Committees.

### Laboratory methods

Collected blood samples were centrifuged for 10 min at 3000 revolutions per min (RPM) within 4 hours of collection. If unable to be centrifuged immediately, blood was stored in a cool environment (fridge or esky) until centrifugation occurred. Following centrifugation, samples were transported on ice to be stored at -80 degree freezer. For samples collected in remote locations, storage for transportation was either on dry ice or in liquid nitrogen ("Biological Shipper", CryoPak Series, Taylor-Wharton, AL, USA). Samples collected on Thursday Island, Queensland were stored at -30 degree for 2-14 days prior to transportation on dry ice to a -80 degree freezer in Cairns.

Serum creatinine and other metabolite assays were performed at each centre as part of standard clinical care. The creatinine assay at each centre was confirmed to have claimed traceability to the Isotope Dilution Mass Spectrometry (IDMS) reference method. The method and instrument used for measurement of each biochemical measure performed at each site are outlined in Table 1.

**Table 1:** Analysis of blood and urine samples

	Instrument used at each Analysis site (with method specified for creatinine and HbA1c)								
**Test**	Royal Darwin Hospital (& Gove, Katherine Hospitals)	Westerns Pathology Darwin (& Katherine)	Westerns Pathology Alice Springs	Alice Springs Hospital (& Tennant Creek Hospital)	Cairns Base Hospital	Royal Perth Hospital	PathWest, Perth	PathWest, Kalgoorlie	PathWest, Broome/Kununurra
Serum creatinine	Enzymatic, Ortho-Clinical Diagnostics, Fusion 5.1	Compensated Jaffe, Roche, Integra 800	Compensated Jaffe, Roche, Integra 400	Enzymatic, Ortho Clinical Diagnostics, Fusion 5.1	Modified Jaffe reaction. Beckman-Coulter DXc600	Jaffe, Abbott, Architect	Jaffe, Abbott, Architect	Enzymatic, Ortho Clinical Diagnostics, V250	Enzymatic, Ortho Clinical Diagnostics, V250
HbA1c	Boronate Affinity, Primus	Immunoassay, Roche, Integra 800	Immunoassay, Roche Integra 400	Immunoassay, Ortho Clinical Diagnostics, Fusion 5.1	Cation Exchange HPLC BioRad Variant II, Nu	Immunoassay, Roche Integra 800	Immunoassay, Roche Integra 800	Pathwest Perth	Pathwest Perth
Lipids	Ortho-Clinical Diagnostics, Fusion 5.1	Roche, Integra 800	Roche, Integra 400	Ortho Clinical Diagnostics, Fusion 5.1	Beckman-Coulter DXc600	Abbott, Architect	Abbott, Architect	Ortho Clinical Diagnostics, V250	Ortho Clinical Diagnostics, V250
Hs-CRP	Normal CRP only	Beckman Immage (Perth)	Beckman Immage (Perth)	Normal only	NA (routine CRP only)	Siemens, BNII	Royal Perth Hospital	Royal Perth Hospital	Royal Perth Hospital
Full blood count	Beckman-Coulter	Beckman-Coulter HmX	Beckman-Coulter MaxM	Beckman-Coulter Hmx or LH500	Sysmex XE5000	Abbott, Cell Dyne Saphire	Beckman-Coulter LH75	Beckman Coulter LH500	Beckman Coulter LH500
Liver function tests	Ortho-Clinical Diagnostics, Fusion 5.1	Roche, Integra 800	Roche, Integra 400	Ortho Clinical Diagnostics, Fusion 5.1	Beckman-Coulter DXc600	Abbott, Architect	Abbott, Architect	Ortho Clinical Diagnostics, V250	Ortho Clinical Diagnostics, V250
Urine creatinine	Ortho-Clinical Diagnostics, Fusion 5.1	Roche, Integra 800	Roche Integra 800 (Perth)	Ortho Clinical Diagnostics, Fusion 5.1	Beckman-Coulter DXc600	Abbott, Architect	Roche Integra 800	Pathwest Perth	Pathwest Perth
Urine albumin	Ortho-Clinical Diagnostics, Fusion 5.1	Roche, Integra 800	Roche Integra 800 (Perth)	Ortho Clinical Diagnostics, Fusion 5.1	Beckman-Coulter DXc600	Abbott, Architect	Roche Integra 800	Pathwest Perth	Pathwest Perth

In addition, serum creatinine will be measured in all samples at Austin Health, using the kinetic Jaffe method and the Roche enzymatic method on a Beckman-Coulter DxC 800 analyser (Fullerton, CA, USA). Beckman-Coulter claims traceability for its Jaffe methods to the IDMS reference method as it does for the Roche enzymatic method. The Roche enzymatic method has been used as a reference method for the revision of the MDRD formula for IDMS aligned assays [28] and has undergone local independent validation [29]. Creatinine will be measured by both the Jaffe and enzymatic methods as many laboratories in Australia measure creatinine with a Jaffe method. This approach will allow for a calibration and standardization of the Jaffe methods against the enzymatic method and assessment of variability caused by the use of different routine methods. Cystatin C will be measured using an automated particle-enhancing immunonephelometric assay on a BN II instrument (Dade Behring, Marburg, Germany). The intra- and interassay CVs for cystatin C were 2.58 and 3.95%, respectively, at a concentration of 1.54 mg/l. Cystatin C measures will be transformed to a GFR equivalent using the formua Cys-GFR = (86.7/cystatin C -4.2). This Cys-GFR method has been validated as an accurate marker of renal function across a wide-range of GFR values using isotopic GFR measurements (plasma dissapearance of ^99 m^Tc-DTPA) as the reference method [18,30].

Iohexol was measured by Austin Health, Melbourne using a validated HPLC assay modified from Niculescu-Duvaz et al [31]. Plasma samples were extracted using a solution of 5% perchloric acid containing internal standard (sodium diatrizoate). Chromatographic separation was achieved using a Microsorb MV C18 5 μ column (Varian Australia) and detection at 244 nm. Analyte concentrations were calculated by comparison to multipoint linear standard curves derived from plasma samples spiked to contain between 3.125 and 800 μg/mL Iohexol (reference material, US Pharmacopoeia). The lower limit of quantitation was 3.125 μg/mL. Intra- and inter-assay performance was assessed using donor plasma spiked with low (20 μg/mL), medium (100 μg/mL) and high (400 μg/mL) concentrations of Iohexol. Precision studies showed a Coefficient of Variation of 1.0% or less, and inaccuracy ± 4.5% or less.

Samples were stored for analysis at a later date for: adiponectin and urine cotinine. For participants of the DXA sub-study, samples have been stored for analysis of 25-hydoxy Vitamin D and parathyroid hormone and bone turnover markers.

### Data handling and statistical methods

At the time of baseline assessment, data was collected on paper forms and then entered into a Microsoft Access database. Data from each site is stored securely at Menzies School of Health Research. All statistical analysis will be performed using the latest available release of Stata software (Stata Corporation, College Station, TX). The predictive performance of formulae methods to estimate GFR will be compared to the reference GFR in terms of precision, bias and accuracy according to the methods of Bland-Altman [32]. The percentage of estimated GFR within 30% of measured GFR will also be determined. If required, new prediction equations will be developed in a "training sample", with their veracity then being tested in a "validation" sample. This will be achieved by randomly dividing the study population into two subgroups, balanced across the five strata. Sample size was determined using the methods of Bland-Altman [32,33] so that the limits of 95% confidence intervals for the mean were equal to pre-specified acceptability limits (a 10% difference in GFR was considered to be acceptable). Sample size was calculated at 600 Indigenous participants across 4 sites (approximately 150 at each site), with considerable heterogeneity in body builds and composition expected both within and between sites. Regression equations will be derived using a similar approach to that used to develop the original MDRD equations [34]. In addition, for the body composition substudy (DXA *vs*. BIA), we will be able to detect a difference ≥ 2% in fat free mass (FFM) estimated by the two methods, with a power >90% based on a sample size of 300, alpha = 0.05 and a CV of 10% in FFM.

Detailed analysis of demographic, clinical, biochemical and socioeconomic factors associated with CKD will involve cross-sectional analysis of the associations between these factors and current GFR. After determining univariate associations between variables, established risk factors and variables with p < 0.2 on univariate analysis will be selected for entry into logistic regression models using the backwards selection method, the outcome variable being current GFR/CKD stage. The degree of coupling of GFR and albuminuria will be determined in participants with type 2 diabetes (after accounting for the use of inhibitors of the renin-angiotensin system). We have recently shown that there is a relatively high prevalence of non-albuminuric renal insufficiency in non-Indigenous participants with type 2 diabetes [35] and preliminary evidence suggests that the prevalence of non-albuminuric renal insufficiency is lower in Indigenous than non-Indigenous diabetic Australians [36].

## Discussion

This study was designed with respect to what is practical and achievable in very remote regions of Northern, Western and Central Australia. The choice of reference measure of GFR was limited by our remote setting and the available time of participants. Nuclear medicine facilities are not available in the vast majority of sites where this project was conducted. Study investigators agreed that 4-hour plasma disappearance of iohexol was the most appropriate reference measure of GFR for the remote setting. The benefits of this reference measure are that it is practical, safe, stable and reproducible [37,38]. In addition, several investigators had experience with use of this measure in the non-Indigenous urban setting (Melbourne) with accurate comparison to 99 mTc-DTPA in participants with diabetes [39]. Based on previous experience of investigators in Indigenous health research, we agreed that a reference measure that required more than 4 hours of participants time would not be practical or achievable.

A potential limitation of our study is the use of Iohexol clearance from serum as the formal GFR measurement as opposed to the use of urinary clearance of iothalamate as the reference method used in the MDRD and CKD-EPI studies [16,34]. Iohexol serum clearance was however used in two studies used in the CKD-EPI validation group [40,41] however the limitation of 4 hours for the studies has the potential to increase inaccuracy in subjects with reduced GFR [40].

The study has encountered several operational challenges to date that have been successfully managed. The first challenge is the significant labour intensity required to recruit each participant and the associated expenses. The proportion of eligible participants approached by the study team, who subsequently consented to and successfully completed the study, varied by site from approximately 30% in urban centres to up to 70% in some remote communities. We managed this challenge by adapting recruitment sites to those that had a higher success rate of obtaining complete data sets and expanding networks and recruitment sites beyond health service providers, e.g. Aboriginal Hostels Limited (provide temporary and transit accommodation in urban and regional centres).

The second challenge encountered by the eGFR study team was to recruit a diverse sample of participants including people living remotely and with poor access to regular medical services. We managed this challenge by: targeting a range of communities or regions; targeting remote-living individuals whilst they were visiting regional centres (Darwin, Katherine and Nhulunbuy: health and non-health service sites); and selection of participants, stratified by study design, using electronic health record data held by urban and remote health services.

The third challenge was to conduct recruitment in such a way to facilitate complete data collection, in particular complete 4 hour reference GFR measures. We commenced recruitment of participants in association with outpatient renal clinics (urban and regional). In the regional setting other commitments for remote participants within the 4-hour duration of the study resulted in non-completion of the reference GFR measures. Reasons included the need to catch transport home (usually a plane) or logistic constraints such as inclement weather conditions. The study recruitment strategy was subsequently changed so that the eGFR study team travelled to remote communities to perform the study in "batches" of 1-3 weeks duration. This strategy was superior in terms of numbers of participants recruited and proportion of participants with complete 4 hour reference GFR measures.

This study should provide evidence for clinical guidelines in a field where evidence is currently lacking: a validated methodology to accurately assess renal function in diverse populations of Indigenous Australians. We expect to determine a validated and practical measure of GFR suitable for use in all Indigenous Australians. This may be cystatin C or eGFR (using MDRD or CKD-EPI formula with/without suitable revision). This measure would enable development of appropriate protocols for investigation and management of CKD and subsequent improvements in clinical practice. We believe that the MDRD- or CKD-EPI derived method (modified as appropriate) is likely to emerge as the cheapest and most widely assessable means of estimating GFR in the immediate future. It seems unlikely that a single correction factor (similar to that for Afro-Americans) is appropriate or practical for Indigenous Australians. However, it may be that a modification of the MDRD or CKD-EPI equation for Indigenous Australians (or for any population) would be to include a measure of fat-free mass. This measure may be obtained by measuring body composition using a simple, portable and inexpensive method such as BIA, once it has been suitably validated against DXA. Thereby this study may improve the utility of eGFR, using a modified MDRD or CKD-EPI formula, across different populations with different body builds and compositions. Our revised formula for eGFR may indeed be of use for assessing kidney function in the broader Australian community, particularly those of variable body builds and/or eGFR>60 ml/min/1.73 m^2^.

## Competing interests

LCW has consults to Impedimed Ltd. Impedimed Ltd. had no involvement, financial or otherwise, in the conception and execution of this study or in the preparation of the manuscript.

All remaining authors declare that they have no competing interests.

## Authors' contributions

LMB drove the design of the study protocol for funding and Ethics applications, coordinated data collection and data management, analysed the data and drafted the manuscript. PL, RM, GJe, AS, KOD conceived of the study and participated in its design. SS was the study manager and participated in study design, ethics applications, data collection and data management. JH participated in study design, data collection and data management. GJo, AE, WH, AC participated in study design. MT, KW, CS participated in the design of the study and provided clinical expertise during the design and data collection phases. LP, LW, RMcD, AB participated in study design. All authors were involved in revising the manuscript for important intellectual content and read and approved the final manuscript.

## Pre-publication history

The pre-publication history for this paper can be accessed here:

http://www.biomedcentral.com/1471-2458/10/80/prepub
